# Advances with Long Non-Coding RNAs in Alzheimer’s Disease as Peripheral Biomarker

**DOI:** 10.3390/genes12081124

**Published:** 2021-07-24

**Authors:** Maria Garofalo, Cecilia Pandini, Daisy Sproviero, Orietta Pansarasa, Cristina Cereda, Stella Gagliardi

**Affiliations:** 1 Genomic and Post-Genomic Unit, IRCCS Mondino Foundation, 27100 Pavia, Italy; maria.garofalo@mondino.it (M.G.); cecilia.pandini@mondino.it (C.P.); daisy.sproviero@mondino.it (D.S.); orietta.pansarasa@mondino.it (O.P.); cristina.cereda@mondino.it (C.C.); 2 Department of Biology and Biotechnology “L. Spallanzani”, University of Pavia, 27100 Pavia, Italy

**Keywords:** long non-coding RNA, Alzheimer’s disease, biomarkers, peripheral system

## Abstract

One of the most compelling needs in the study of Alzheimer’s disease (AD) is the characterization of cognitive decline peripheral biomarkers. In this context, the theme of altered RNA processing has emerged as a contributing factor to AD. In particular, the significant role of long non-coding RNAs (lncRNAs) associated to AD is opening new perspectives in AD research. This class of RNAs may offer numerous starting points for new investigations about pathogenic mechanisms and, in particular, about peripheral biomarkers. Indeed, altered lncRNA signatures are emerging as potential diagnostic biomarkers. In this review, we have collected and fully explored all the presented data about lncRNAs and AD in the peripheral system to offer an overview about this class of non-coding RNAs and their possible role in AD.

## 1. RNA Metabolism in Alzheimer’s Disease

Alzheimer’s disease (AD) is a progressive neurodegenerative disorder that leads to intellectual functions’ impairment. AD is the most common type of dementia in aging populations causing neuropathology in specific brain regions, including hippocampus, amygdala, and frontal and temporal cortices. Complex multifactorial interactions among genetic, epigenetic, and environmental components contribute to AD onset. Although much emphasis has been placed on the role of protein aggregates (Aβ plaques and tau tangles) in AD, recent multiple lines of evidence converge on altered RNA metabolism as a contributing factor in the pathogenesis of this disorder. In particular, non-coding RNAs’ role is emerging as involved in pathogenesis, diagnosis and therapy of AD. For instance, many microRNAs (miRNAs) have been identified as key elements for the regulation of memory process and cognitive functions lost in AD [1]. They can act through the regulation of activity-mediated protein synthesis at the synaptic level [2], the regulation of Aβ production [3,4] and tau phosphorylation [3]. Circular RNAs (circRNAs), a type of single-stranded RNA which forms a covalently closed continuous loop, can act as a miRNA “sponge” to quench normal miRNA functions [5]. This mechanism has been found also in AD, where the altered circRNA ciRS-7 sponging activity for miRNA-7 leads to the lack of essential proteins for the clearance of amyloid peptides in AD brain [6]. Moreover, mounting evidence shows that long non-coding RNAs (lncRNAs) are aberrantly expressed in AD progression and participate in the regulation of Aβ peptide [7,8] tau [9], inflammation and cell death [10,11].

## 2. Long Non-Coding RNAs

LncRNAs are defined as non-coding RNA molecules longer than 200 nucleotides. Most of them are transcribed by RNA polymerase II and are often post transcriptionally modified by splicing, 5′ 7-methylguanosine capping and a 3′ polyadenylation; however, they lack coding capacity [12]. Human GENCODE suggests that the human genome contains more than 16,000 lncRNA genes, but other estimates exceed 100,000 human lncRNAs [13]. Despite not being translated into proteins, lncRNAs are functional molecules with high heterogeneity and functional versatility that relies on their ability as long RNA molecules to conform to different structures and molecular interactions. Indeed, lncRNAs can regulate, among other things, transcriptional regulation in cis or trans, organization of nuclear domains, and regulation of proteins or RNA molecules, affecting numerous biological and pathological processes [14].

## 3. lncRNAs in AD Peripheral System

### 3.1. Blood

The discovery of peripheral biomarkers for neurodegenerative disease, such as AD, is needed. LncRNAs may be a noninvasive target to confirm AD diagnosis and they can also be used as prognostic biomarkers.

Different papers have investigated lncRNAs in blood for AD patients. Kurt and collaborators [15] have investigated lncRNAs’ expression difference between AD patients and controls in peripheral blood mononuclear cell (PBMC) by microarray analysis. Their data showed that 34 lncRNAs have been found deregulated, in particular the most altered lncRNA is an antisense transcript named TTC39C-AS1. This antisense is interesting since its sense gene, *TTC39C*, is involved in neurogenic atrophy [16]. Next, another highly deregulated lncRNA was LOC401557 that is an uncharacterized lncRNA very abundant in the brain tissue [17]. Gene deregulation generally implicates changes in gene expression altering cell homeostasis, and its understanding may provide new insights into the mechanisms involved in human diseases [18]. In general, different pathways in which lncRNA may have a role have been identified, such as amyloidogenic and mTOR pathways. For both, a deregulation of lncRNAs occurs as LINC01503 and LINC01420 are altered in PBMCs and also in brain [19,20].

We previously demonstrated deregulated lncRNAs in PBMCs from AD patients by RNA-seq. We compared the lncRNA profile of AD patients with two other neurodegenarative diseases, Parkinson’s disease and amyotrophic lateral sclerosis [21]. The data showed that CH507-513H4.4, CH507-513H4.6, CH507-513H4.3 lncRNAs are deregulated in AD PBMC compared to controls. They are novel transcripts, similar to YY1 Associated Myogenesis RNA 1 (YAM1), and they are reported as AD associated in the LncRNADisease v2.0 Database [22]. These lncRNAs were specific for AD-in fact, no deregulation was found in the other diseases. Moreover, lncRNA pathway analysis was performed using the LncPath R package that showed an involvement of Mapk signaling, cytokine receptor interaction, chemokine signaling, natural killer cell mediated cytotoxicity and regulation of actin cytoskeleton. 

### 3.2. Plasma

Two main plasma lncRNAs have been proposed as possible AD biomarkers: BACE1-AS and 51A [23].

51A is the antisense transcript of *SORL1* gene that was described as associated to AD for the first time in 2004, but its role is not clear [24]. SORL1 is involved in APP processing and trafficking. It may bind newly made Aβ in the neuron and steers it toward lysosomes, where it is degraded [25,26]. Besides this, SORL1 as an ApoE receptor is likely to participate in the lipid metabolism of AD genesis [27].

SORL1-AS (51A) expression leads to Aβ-42 accumulation, and it has been found to be increased in plasma and brain of AD patients compared to controls [28]. Clinical correlation showed that lncRNA 51A was negatively correlated with the Mini-Mental State Examination (MMSE) scores in AD patients.

About LncRNA BACE1-AS, its plasma level in AD patients was significantly higher compared to controls [29], while there was no correlation with MMSE scores. On the other hand, it has recently been demonstrated that lncRNA BACE1-AS may discriminate between full AD and controls but also between pre-AD and controls, suggesting that lncRNAs could be a predictive biomarker [30]. BACE1-AS regulates BACE1 mRNA and protein expression and may also increase BACE1 stability [8]. In fact, when BACE1-AS is silenced, the activity of BACE1 mRNA is attenuated and the production of Aβ-42 oligomers is reduced [31].

### 3.3. Extracellular Vesicles (EVs)

The presence of lncRNAs is also observed in extracellular vesicles (EVs). EVs are heterogenous lipid bound vesicles that are released and circulate in the extra-cellular space [32]. The two main subtypes of EVs are microvesicles (MVs), mostly derived from plasma membrane and 100–500 nm in diameter, and exosomes, generated through the classical endosome-multivesicular body (MVB) pathway and 30–150 nm in diameter [33]. The International Society for Extracellular Vesicles (ISEV) has updated EVs’ nomenclature, defining as small EVs (SEVs) particles that are <100 nm or <200 nm and large EVs (LEVs) those that are >200 nm [33].

LncRNAs have mostly been observed packaged into SEVs [34,35]. SEVs can be released by practically all eukaryotic cells [36]. We found two studies concerning lncRNAs in AD in SEVs derived from plasma and cerebrospinal fluid (CSF).

BACE1-AS transcript was measured in plasma-derived SEVs from 72 AD and 62 controls. The level of this transcript was different in the two groups, being significantly higher in AD patients [37]. This result is in contrast with a previous study, that analyzed a smaller cohort of subjects, where the level of BACE1-AS remained unchanged in AD plasma SEVs [30].

BACE1-AS is able to influence the expression of Aβ and is described in AD pathogenesis [38]. Given the need of improving accuracy of AD diagnosis, Wang and collaborators tried to link pathological changes in the brain and the altered expression of BACE1-AS. However, they found no correlation between this lncRNA and Magnetic Resonance Imaging (MRI) data. Nevertheless, they also performed a receiver operating characteristic (ROC) curve analysis, which is a graphical approach for comparing the relative performance of different classifiers and to determine whether a classifier performs better than random guessing [39]. They demonstrated that when exosomal BACE1-AS levels are combined with the volume and thickness of the right entorhinal cortex, specificity and sensitivity were at high percentage, making these parameters potential biomarkers of AD [37].

The expression of two lncRNAs, RP11-462G22.1 and PCA3, was also evaluated in CSF-derived SEVs from AD patients [40]. These two transcripts were found to be associated with Parkinson’s disease (PD). These lncRNAs may not represent the perfect biomarkers for discriminating AD and PD, due to the fact that they are deregulated in both conditions, but they could rather be used as indicative molecules for neurodegeneration. RP11-462G22.1, instead, was found to be highly expressed in AD and PD. It is a muscular dystrophy-associated lncRNA that was predicted to be the target of 21 microRNAs, making it a potential competing endogenous RNA (ceRNA) [41]. PCA3, another lncRNA up-regulated in CSF-derived SEVs from AD patients, may be targeted by 14 microRNAs [42]. PCA3’s biological function in neurodegenerative disorders is still unknown.

So far, the study of lncRNAs in EVs from AD patients is not sufficient for providing informative evidence of their role in the pathogenesis of this disease. Nor has a relevant screening of these molecules been published in order to highlight reliable biomarkers that could be used in the diagnosis or prognosis of AD.

### 3.4. Cerebrospinal Fluid (CSF)

The most instructive fluid in biomarker detection for neurodegeneration is cerebrospinal Fluid (CSF) [43]. Thus, we explored literature in order to highlight the most promising lncRNAs studied in CSF of AD patients.

MALAT1, a long intergenic non-coding RNA, regulates synaptogenesis and, in fact, its expression is widely observed in neurons [44]. It may be used as a diagnostic biomarker of AD in CSF, where it was found down-regulated [45]. The role of MALAT1 was initially described in AD models where the expression of the transcript was both up and down-regulated [11]. In this study, enhanced neuron apoptosis, repressed neurite outgrowth and elevated inflammation-related molecules were observed where MALAT1 levels were lower. Moreover, they found miR-125b, which induces the processes listed above, to be negatively affected by MALAT1. Thus, low levels of lncRNA MALAT1 promote miR-125b enrichment, which in turn increases prostaglandin-endoperoxide synthase 2 (*PTGS2*) and cyclin-dependent kinase 5 (*CDK5*) expression levels and decreased forkhead box Q1 (*FQXQ1*). Interestingly, the intercorrelation of MALAT1 and miR-125b with *FOXQ1*, *PTGS2* and *CDK5* was also confirmed in CSF of AD patients [46]. In addition to functional characterization, this lncRNA–miRNA axis in CSF was also used for predicting Mini-Mental State Examination (MMSE) score decline at 1 year, 2 years and 3 years in AD patients.

Glial cell-derived neurotrophic factor (GDNF) is involved in neurite branching and synaptic plasticity [47]. In CSF of AD patients, GDNF mRNA is highly up-regulated [48]. The identification of a cis-antisense non-coding RNA to GDNF (GDNF-AS1 or GDNFOS) and its dependence to GDNF expression led Airavaara and collaborators to speculate that GDNF-AS1 may also be involved in synaptic plasticity and that further studies are needed to demonstrate the implication of this lncRNA in AD pathogenesis [47].

Long non-coding RNA activated by TGF-beta (lncRNA-ATB), firstly identified in 2014 [49], is abnormally expressed in central nervous system cancers [50]. Its expression was also altered in CSF of AD patients, where it was highly increased [51]. For this reason, deregulation of lncRNA-ATB may be used as a hallmark of disease rather than a specific biomarker. Moreover, in a recent study adult malignant brain tumors and AD were found to share some environmental risks [52]. LncRNA-ATB is indeed up-regulated both in AD patients and in glioma tumors.

To study the effect of lncRNA-ATB up-regulation, Wang and collaborators used PC12 cells and discovered that miR-200 is negatively affected by this lncRNA. MiR-200 in turn inversely regulates makorin ring finger protein 3 (MKRN3 or ZNF127), which is a 3-ubiquitin ligase potentially affecting gene expression and targeted protein degradation [47]. The inhibition of miR-200 mediated by lncRNA-ATB overexpression aggravated PC12 cells injury induced through Aβ25-35 [51]. However, the role of ZNF127 in neurodegeneration remains unclear. Altogether, these results highlight the relevance of the lncRNA–ATB/miR-200 axis in AD (Table 1).

## 4. Conclusions

In this review, we have explored literature classifying the current knowledge about lncRNAs in peripheral tissue of Alzheimer’s disease patients. We found that several non-coding transcripts have been identified as potential biomarkers of this disease. Moreover, some studies have also highlighted the need to characterize the functional role of these molecules in the pathogenesis of Alzheimer’s. In particular, the up-regulation of BACE1-AS in different tissues from AD patients appears to be a promising lncRNA in the study of AD due to its involvement in β-secretase regulation.

In conclusion, research concerning lncRNAs in neurodegenerative pathogenesis needs to be implemented in the future, covering both their potential as biomarkers and as therapeutic targets.

## Figures and Tables

**Table 1:** Deregulated lncRNA in peripheral tissue of AD patients.

Deregulated lncRNA in AD	Trend	Source	Reference	Tissue Expression
TTC39C-AS1	up-regulated	Blood	[1,2,3,4,5,6]	adrenal; brain; breast; lymphnode; testes; thyroid
LOC401557	up-regulated	Blood	[17]	adipose; adrenal; brain; breast; colon; foreskin; heart; HLF; kidney; liver; lung; lymphnode; ovary; placenta; prostate; skeletal muscle; testes; thyroid; WBC
CH507-513H4.4	up-regulated	Blood	[21]	/
CH507-513H4.6	up-regulated	Blood	[21]	/
CH507-513H4.3	up-regulated	Blood	[21]	/
SORL1-AS (51A)	up-regulated	Plasma	[28]	/
BACE1-AS	up-regulated	Plasma	[30]	brain; ovary; testes; thyroid
BACE1-AS	up-regulated	Plasma SEVs	[37]	brain; ovary; testes; thyroid
RP11-462G22.1	up-regulated	CSF SEVs	[40]	adipose; adrenal; brain; breast; colon; foreskin; heart; HLF; kidney; liver; lung; lymphnode; ovary; placenta; prostate; skeletal muscle; testes; thyroid; WBC
PCA3	up-regulated	CSF SEVs	[40]	brain; HLF; kidney; lymphnode; ovary; prostate; testes
MALAT1	down-regulated	CSF	[45]	adipose; brain; breast; lymphnode; prostate; testes; thyroid
lncRNA-ATB	up-regulated	CSF	[51]	adrenal; brain; breast; heart; HLF; liver; ovary; testes; thyroid

Deregulated lncRNAs in AD patients are reported together with their trend (up or down-regulated), their biofluid source and the corresponding literature reference. Using NONCODE database (www.noncode.org), human tissue where relative lncRNA was detected is reported. Human lung fibroblast (HLF); White blood cells (WBC).
